# Insulin Resistance in Chileans of European and Indigenous Descent: Evidence for an Ethnicity x Environment Interaction

**DOI:** 10.1371/journal.pone.0024690

**Published:** 2011-09-08

**Authors:** Carlos A. Celis-Morales, Francisco Perez-Bravo, Luis Ibañes, Ruth Sanzana, Edison Hormazabal, Natalia Ulloa, Carlos Calvo, Mark E. S. Bailey, Jason M. R. Gill

**Affiliations:** 1 Institute of Cardiovascular and Medical Sciences, College of Medical, Veterinary and Life Sciences, University of Glasgow, Glasgow, United Kingdom; 2 School of Life Sciences, College of Medical, Veterinary and Life Sciences, University of Glasgow, Glasgow, United Kingdom; 3 Laboratory of Nutritional Genomics, Department of Nutrition, Faculty of Medicine, University of Chile, Santiago, Chile; 4 Center of Nutrition, Metabolism and Physical Activity (CNEF), Concepcion, Chile; 5 Department of Clinical Biochemistry and Immunology, Universidad de Concepción, Concepción, Chile; Universidad Peruana Cayetano Heredia, Peru

## Abstract

**Background:**

Effects of urbanisation on diabetes risk appear to be greater in indigenous populations worldwide than in populations of European origin, but the reasons are unclear. This cross-sectional study aimed to determine whether the effects of environment (Rural vs. Urban), adiposity, fitness and lifestyle variables on insulin resistance differed between individuals of indigenous Mapuche origin compared to those of European origin in Chile.

**Methodology/Principal Findings:**

123 Rural Mapuche, 124 Urban Mapuche, 91 Rural European and 134 Urban European Chilean adults had blood taken for determination of HOMA-estimated insulin resistance (HOMA_IR_) and underwent assessment of physical activity/sedentary behaviour (using accelerometry), cardiorespiratory fitness, dietary intake and body composition. General linear models were used to determine interactions with ethnicity for key variables. There was a significant “ethnicity x environment” interaction for HOMA_IR_ (Mean±SD; Rural Mapuche: 1.65±2.03, Urban Mapuche: 4.90±3.05, Rural European: 0.82±0.61, Urban European: 1.55±1.34, *p*_(interaction)_ = 0.0003), such that the effect of urbanisation on HOMA_IR_ was greater in Mapuches than Europeans. In addition, there were significant interactions (all *p*<0.004) with ethnicity for effects of adiposity, sedentary time and physical activity on HOMA_IR,_ with greater effects seen in Mapuches compared to Europeans, an observation that persisted after adjustment for potential confounders.

**Conclusions/Significance:**

Urbanisation, adiposity, physical activity and sedentary behaviour influence insulin resistance to a greater extent in Chilean Mapuches than Chileans of European descent. These findings have implications for the design and implementation of lifestyle strategies to reduce metabolic risk in different ethnic groups, and for understanding of the mechanisms underpinning human insulin resistance.

## Introduction

Prevalence of diabetes is increasing worldwide, but differs widely between regions [1], which can be partly attributed to differences in urbanisation and obesity [2]. However, differences in environment alone do not appear to tell the whole story, particularly in elucidating why certain populations and ethnic groups experience a disproportionately high prevalence of type 2 diabetes when they adopt a Western lifestyle. The classic example of this is the Pima Indians, who when living a traditional rural lifestyle in Mexico are lean, active and have low diabetes prevalence, but when living in the US, are generally obese and have a diabetes prevalence in the adult population of ∼40% [3]. This pattern is also evident in other indigenous populations throughout the Americas and Australasia [4].

Mapuches – an indigenous Native American population from Chile – appear to follow this pattern of a disproportionate increase in risk of diabetes (compared to Chileans of White European descent) when they transition from a traditional rural lifestyle to an urban one. Limited data, based on observations from small sample groups, suggest that diabetes prevalence for Mapuches living in traditional rural environments is low, at between 1% and 4% of the adult population [5], [6], but rises to 6.3–8.2% amongst urban-dwelling Mapuches [7], [8]. In contrast, Chileans of European descent (∼92% of the Chilean population [9]) have a smaller differential between the two environments, with higher rates of diabetes than Mapuches in rural areas (4.5%) and lower rates in urban (5.8%) settings [10]. Interestingly, differences in BMI do not explain the difference in diabetes prevalence between rural and urban Mapuches [5], [8], which contrasts with observations from other indigenous groups where the transition to an urban environment is associated with a substantial increase in obesity prevalence [3], [11], [12]. It is likely that, in common with other indigenous groups, Mapuches have innate factors that predispose them to increased insulin resistance and diabetes risk, whose effects are revealed on adoption of an urbanised lifestyle. However, it is also possible that Mapuches have a greater lifestyle shift compared to European Chileans when moving from rural to urban settings, which could explain the larger apparent effect of environment on diabetes risk in this group. For example, physical activity or cardiorespiratory fitness levels both predict diabetes risk independently of obesity [13], [14]: larger differences in these factors or in aspects of body composition not reflected in BMI measurements may be evident between urban and rural Mapuches compared to Europeans living between these two environments. Thus, in order to determine whether Mapuches are indeed more susceptible to the adverse metabolic effects of an urban environment than European Chileans, detailed and objective measures of physical and lifestyle variables of both Mapuches and European Chileans living in rural and urban environments are required.

The aims of the present study were therefore twofold. The first aim was to determine cross-sectionally, using objective measures, whether urbanisation influenced metabolic risk factors, cardiorespiratory fitness, body composition and lifestyle variables differently in Chileans of Mapuche compared to European descent. The second aim was to determine whether physical activity, sedentary behaviour, cardiorespiratory fitness, dietary intake and body composition affected insulin resistance differently between Mapuche and European Chilean populations.

## Methods

### Ethics statement

All participants gave written informed consent prior to inclusion in this study, which was approved by Research Ethics Committees at the University of Glasgow, University of Chile, and University of Concepcion.

### Participants and study design

The sampling strategy was cross-sectional in design and involved the planned collection of data from four groups of participants: Mapuches living in Rural and Urban environments and Chileans of European descent living in Rural and Urban environments. Participants of both sexes, aged 20–60 years, were recruited, from towns/cities and from rural villages and communities in three regions of Chile – Los Ríos, Bio-Bio and Metropolitana – between February and June 2008. The study was advertised using posters displayed in prominent locations (in the smaller communities), using local radio advertisements, and by open invitations to the membership of local community organisations, social and sports clubs. These methods were employed in an attempt to ensure that all groups within each society had an approximately equal chance of seeing the study and of signing up to it, thereby minimising potential recruitment bias. In the smaller communities, particularly those rural communities dominated by Mapuches, almost all adults in the community were sampled after agreement from local community leaders to let us advertise the study locally. Sampling rates were lower in larger communities.

To ensure that Mapuche and European population groups with as little admixture as possible were studied, the following inclusion criteria were applied: i) Mapuche participants were included if they had; a) both maternal and paternal last names of Mapuche origin; these names are identifiably different from European names, b) both parents of Mapuche origin, c) type O blood group in the ABO blood group system (Mapuches have a very high frequency (95%) of this blood group [8], thus possession of A or B blood groups is likely to indicate mixed ancestry). ii) Europeans were included if they had a) both maternal and paternal last names of European origin, b) both parents of European origin. Individuals with a known history of cardiovascular disease or taking anti-hypertensive or diabetes medications were excluded from participation. All participants were told that personal health-related phenotypic data would be communicated back to them via their general practitioner, but they received no other incentive to participate.

### Physical, biochemical and behavioural measurements

All phenotype measurements were conducted ‘in the field’ at the locations where the study populations resided. Facilities for centrifuging blood samples and for cooling/freezing blood and plasma were transported to all locations including remote rural communities. Height, body mass, waist and hip circumferences and skinfolds at four sites (biceps, triceps, subscapular, suprailiac) were measured using standard protocols [15]. Body composition was calculated from skinfold measures [16]. Blood pressure was measured on the right arm after at least 10 minutes of seated rest using an automated blood pressure monitor (Omron HEM705 CP, Omron Healthcare UK Limited, Milton Keynes, UK) which has been validated according to the European Society of Hypertension International Protocol [17]. The mean of two blood pressure readings was used in analysis.

The Chester step-test, a validated 3-stage incremental stepping test, was used to estimate maximal oxygen uptake (VO_2max_) [18] as a measure of fitness. Participants wore accelerometers (ActiTrainer; ActiGraph LLC, Pensacola, FL, USA) on the left hip at all times, except when showering, swimming and sleeping, for seven consecutive days to objectively assess physical activity levels. Dietary intake was assessed by 7-day weighed food record and analyzed using the Chilean Food Composition Database (Software MINUTA, University of Concepcion, Chile).

Venous blood samples were drawn after an overnight fast and collected into potassium EDTA tubes and placed on ice. Plasma was separated within 10 minutes of collection and frozen at −20°C until analysis. Glucose, triglyceride (TG), total cholesterol, HDL cholesterol, γ- glutamyltransferase (GGT), alanine aminotransferase (ALT) and C-reactive protein (hsCRP) concentrations (using a high sensitivity assay) were determined by enzymatic colorimetric methods using commercially available kits (Roche Diagnostics Gmbh, Mannheim, Germany; Randox Laboratories Ltd., Co. Antrim, Ireland; and Kamiya Biomedical, Seattle, USA). LDL cholesterol was calculated using the Friedewald equation [19]. Insulin and leptin concentrations were determined by radioimmunoassay (Diagnostic System Labs, TX, USA and Linco Research Inc, St. Louis MO, USA). Coefficients of variation were <3.0% for all enzymatic colorimetric assays, 5.0% for insulin and 3.1% for leptin.

Participants' health history, including smoking status and family health history, was determined by questionnaire. Socioeconomic status was determined with the European Society for Opinion and Marketing Research (ESOMAR) questionnaire validated in the Chilean population [20]. The original 6 ESOMAR socioeconomic classes were re-grouped into three classes by combining the two lower, two middle and two higher classes for analysis. Demographic and cultural data (age, attained education, most recent occupation, and ethnicity) were determined using the Chilean Socioeconomic Characterisation Questionnaire [21]. All questionnaire data were collected during in-person interviews.

### Data and statistical analysis

Data on which to base a sample size calculation for this study were limited as there have been few previous investigations of insulin resistance in Mapuches. The available data, based on small samples, indicated that the population SD for HOMA_IR_ was ∼3–4 units in Mapuches [8], and <1 unit in the non-obese urban Chilean general population [22]. Based on this, we estimated an overall population SD for HOMA_IR_ of ∼3 units, and predicted that the study would need power to detect mean group differences of ∼2 HOMA_IR_ units. Power calculations using Minitab (version 14, Minitab Inc., State College, PA, USA) indicated that 65 participants in each of the four study groups would provide 80% power to detect such differences in HOMA_IR_ between groups at the *p*<0.004 level (see below for adjustment of the critical *p*-value to account for multiple testing). This was therefore set as our minimum recruitment target. The upper limit for participant numbers was determined by participant availability within the data collection time-frame.

Accelerometer readings were summarized in 60-second epochs and Freedson cut-points used to define intensity domains [23]. Data from participants with at least 10 hours of daily accelerometer wear time for 4 days were included in the analysis. Non-wear was defined by intervals of at least 60 minutes of zero activity counts [24]. Wear time was calculated by subtracting non-wear time from 24 hours. Activity count values of <100 count.min^−1^ were defined as sedentary behaviour [25]. Insulin resistance was assessed using the Homeostasis Model Assessment of Insulin Resistance (HOMA_IR_) [26].

Data were analyzed using Statistica (version 8.0; StataSoft, Tulsa, USA) and Minitab. Quantitative data were tested for normality using the Anderson–Darling normality test, subjected to Box-Cox analysis, and transformed as appropriate. Accordingly, statistical analysis for insulin, HOMA_IR,_ GGT, ALT and hsCRP was performed using logarithmically transformed data.

To determine whether the effects of urbanisation differed between Chileans of European and Mapuche descent, anthropometric, metabolic, fitness, physical activity and dietary variables were compared between the Rural Mapuche, Urban Mapuche, Rural European and Urban European groups using General Linear Models, considering ethnicity and environment main effects and the ethnicity x environment interaction. In cases where this model revealed no significant ethnicity x environment interaction, the ethnicity and environment main effects were subsequently determined in a model without the interaction term. Men and women were combined in these analyses; all analyses (except age) were undertaken on age- and sex-adjusted data. Missing data values were not imputed. Numbers of observations for individual variables are presented as supplementary data (Table S1).

To determine whether the effects of adiposity, physical activity, fitness and dietary intake on insulin resistance differed between Mapuches and Europeans, participants were divided into sex-specific tertiles for adiposity, physical activity, fitness and dietary intake variables (see Table S2 for tertile cut-points), and General Linear Models were used to determine main effects of increasing ‘tertile’ of each variable on HOMA_IR,_ and any ethnicity x tertile interactions. Tertiles for men and women were combined in this analysis (i.e. Tertile 1 in the analyses contained the lowest third of women and the lowest third of men for any given variable). Rural and Urban groups were combined in these analyses. All models were adjusted for age and sex and further models adjusted for potential confounding variables were undertaken as appropriate.

The critical (α-value) for *p*, at which statistical significance was accepted, was corrected for multiple testing using the Bonferroni method as implemented at the Simple Interactive Statistical Analysis website (http://www.quantitativeskills.com/sisa/), taking into account the number of comparisons made (n = 36) and the mean correlation between the variables tested (r = 0.28). Accordingly, statistical significance was accepted at *p*<0.004 for all analyses.

## Results

A total of 873 individuals (247 Rural Mapuche, 187 Urban Mapuche, 216 Rural European, 223 Urban European) responded to our call for volunteers. Of these, 472 individuals (123 Rural Mapuche, 124 Urban Mapuche, 91 Rural European, 134 Urban European) fulfilled the inclusion/exclusion criteria and agreed to participate in the study. Of the 401 individuals who responded but were not included, 291 did not meet the inclusion/exclusion criteria and 110 chose not to participate. The number of eligible non-participants was higher in Mapuches (62 Rural, 11 Urban) than Europeans (28 Rural, 9 Urban): this was largely the consequence of religious or ethnically-influenced political concerns about the study in some members of the Rural Mapuche population. Three Urban Europeans, 1 Rural European, 11 Urban Mapuches and 4 Rural Mapuches had fasting glucose concentrations >7.0 mmol.l^−1^ in the blood sample collected, indicating possible undiagnosed diabetes. These participants were included in the data analyses reported below, however excluding these individuals from the analyses did not alter any of the study findings (data not shown). Demographic data, by sub-group (Rural Mapuche, Urban Mapuche, Rural European and Urban European) are shown in Table 1. In the sample taken for this study, Mapuches were slightly younger overall than the Europeans (by 3.5 years, *p* = 0.003). Because of this difference, all subsequent statistical analyses were performed on age-adjusted (and sex-adjusted) data.

**Table 1:** Demographic and anthropometric variables by ethnic group and environment.

	Mapuche		European		*p* value	
	Rural	Urban	Rural	Urban	Ethn	Env
**Demographic Variables**						
N (men / women)	54 / 69	45 / 79	36 / 55	42 / 92	-	**-**
Age	36.7 ±11.9	34.1±12.5	40.9±13.7	37.5±12.4	**0.003**	0.014
Smoking Status(Never / Ex / Current)	80 / 16 / 27	50 / 25 / 49	50 / 16 / 25	44 / 36 / 54	-	-
Socio economic Level(Lower / Middle / Higher)	95 / 21 / 7	24 / 65 / 35	38 / 33 / 20	32 / 42 / 60	-	-
Education Level(Primary / Secondary / Tertiary)	94 / 18 / 11	10 / 83 / 31	30 / 40 / 21	7 / 52 / 75	-	-
**Anthropometric variables**						
Normal / Overweight / Obese ^a^	18 / 51 / 54	39 / 55 / 30	17 / 41 / 33	48 / 47 / 39	**-**	-
Body mass (kg)	75.8±11.8	73.3±12.5	73.9±13.3	71.2±13.1	0.327	0.018
Height (m)	1.57±6.9	1.61±8.6	1.59±9.4	1.59±8.5	0.007	0.126
BMI (kg.m^-2^)	30.5±4.3	28.4±4.8	29.2±4.9	28.2±5.4	0.008	**0.002**
Waist (cm)	106.9±11.5	102.1±12.8	104.2±14.5	101.8±15.7	0.155	0.012
Hip (cm)	117.2±11.1	113.3±10.7	114.9±13.0	112.2±13.1	0.042	0.008
Body fat (%)	29.4±5.3	31.2±5.2	28.6±6.1	31.5±6.0	0.021	**0.0001**

Data are presented as mean ± SD for continuous variables or as numbers of individuals in each category for categorical variables. Statistical analysis (except for age) was undertaken on age- and sex-adjusted data. For continuous variables, *p* values shown are for comparisons of means between Rural Mapuche, Urban Mapuche, Rural European and Urban European groups for main effects of ethnicity (Ethn) and of environment (Env). Results of further models assessing the Ethnicity x environment (Ethn x Env) interaction effect are not shown as interaction terms in all the models were non-significant (all *p*>0.28). Significant *p* values (i.e. *p*<0.004) are shown in bold. ^a^Participants were classified into BMI categories using the standard cut-offs: normal (<25.0 kg/m2), Overweight (25.0–29.9 kg/m2) or Obese (>30.0 kg/m2). No formal comparisons between groups were made for the categorical variables.

### Anthropometric variables for Mapuches and Europeans living in Rural and Urban Environments

Table 1 displays anthropometric variables for the four sub-groups along with the results of tests for differences based on ethnicity and/or environment. No significant ethnicity x environment interactions were observed for any of these variables. However, significant main effects of environment were evident for BMI and percentage body fat, with the Rural participants having higher mean BMI (by 1.6 kg.m^−2^, *p* = 0.002) but lower mean body fat measurements (by 2.2 percentage points, *p* = 0.0001) than their Urban counterparts. This indicates that the higher BMI observed in the Rural participants was a consequence of greater fat-free mass.

### Metabolic variables for Mapuches and Europeans living in Rural and Urban Environments

**Figure 1:**
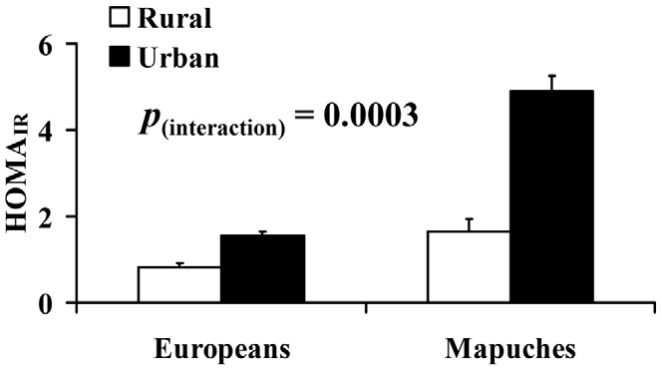
Effect of ethnicity and environment on HOMA_IR_ in European and Mapuche participants. Bars show mean±SEM for all groups. *p*_(interaction)_ describes the ‘ethnicity x environment’ interaction for HOMA_IR_ in age- and sex-adjusted analysis.

Table 2 shows metabolic variables for the four sub-groups. There was a significant ‘ethnicity x environment’ interaction for HOMA_IR_ (*p* = 0.0003), such that HOMA_IR_ was 3-fold higher in Urban compared to Rural Mapuches but only 1.9-fold higher in Urban compared to Rural Europeans (Table 2, Figure 1). In addition to this interaction effect, HOMA_IR_ was higher in Mapuches than Europeans (*p* = 0.0001) and was higher in Urban compared to Rural groups (*p* = 0.0001). A similar pattern was observed for fasting insulin concentrations.

**Table 2:** Metabolic variables by ethnic group and environment.

	Mapuche		European		*p* value		
	Rural	Urban	Rural	Urban	Ethn	Env	Ethn x Env Interaction
Systolic Blood Pressure (mmHg)	120.3±15.3	124.3±17.9	120.7±16.7	123.7±16.9	0.356	**0.001**	0.948
Diastolic Blood Pressure (mmHg)	76.6±11.8	75.7±12.6	75.5±12.0	75.8±11.9	0.300	0.776	0.503
Glucose (mmol.l^−1^)	5.28±1.21	5.42±1.40	5.26±0.93	5.79±1.03	(0.428)	**0.001**	0.049
Insulin (pmol.l^−1^)^a^	6.7±8.5	20.7±15.8	3.4±2.3	5.9±4.6	**(0.0001)**	**(0.0001)**	**0.0002**
HOMA_IR_^a^	1.65±2.03	4.90±3.05	0.82±0.61	1.55±1.34	**(0.0001)**	**(0.0001)**	**0.0003**
Triglyceride (mmol.l^−1^)	1.05±0.45	1.36±0.68	1.21±0.54	1.23±0.75	0.569	**0.0005**	0.154
Total cholesterol (mmol.l^−1^)	4.51±1.00	4.76±1.27	4.73±1.33	4.79±1.20	0.868	0.019	0.554
HDL cholesterol (mmol.l^−1^)	1.08±0.40	0.90±0.37	0.95±0.40	0.90±0.36	0.607	**0.0001**	0.170
LDL cholesterol (mmol.l^−1^)	2.95±1.08	3.24±1.37	3.23±1.42	3.32±1.30	0.900	0.009	0.520
Leptin (ng.ml^−1^)	9.79±8.0	19.4±14.2	8.92±7.04	13.2±14.0	**0.0007**	**0.0001**	0.026
GGT (U.L^−1^)^a^	31.3±19.1	50.4±41.7	27.1±17.4	30.7±24.4	**0.0001**	**0.0001**	0.099
ALT (U.L^−1^)^a^	3.60±0.65	3.37±0.65	3.45±0.55	3.36±0.68	0.159	0.061	0.186
HsCRP (mg.l^−1^)^a^	1.04±1.08	1.43±1.56	1.23±1.22	1.56±1.48	0.416	**0.002**	0.612

Data are presented as mean ± SD. Statistical analysis was undertaken on age- and sex-adjusted data. *p* values shown are for comparisons of means between Rural Mapuche, Urban Mapuche, Rural European and Urban European groups for main effects of ethnicity (Ethn) and of environment (Env), and the Ethnicity x environment (Ethn x Env) interaction effect: for the main effects, those in parentheses indicate residual main effects in a model with a significant interaction term, while those not in parentheses indicate main effects in a model without the interaction term. Significant *p* values (i.e. *p*<0.004) are shown in bold. ^a^ Statistical analyses performed on log-transformed data.

No significant ‘ethnicity x environment’ interactions were observed for any of the other measured metabolic variables, however main effects for ethnicity or environment were observed for some factors. Systolic blood pressure, and glucose, triglyceride, leptin, GGT and hsCRP concentrations were higher, and HDL cholesterol concentration was lower, in Urban compared to Rural groups (all *p*<0.004). In addition, leptin and GGT concentrations were higher in Mapuches compared to Europeans (both *p*<0.004).

### Fitness and physical activity for Mapuches and Europeans living in Rural and Urban Environments

Fitness and physical activity variables for the four sub-groups are shown in Table 3. A significant ethnicity x environment interaction was evident for VO_2max_ (*p* = 0.0001), such that the difference in VO_2max_ between Rural and Urban Mapuches (8.6 ml.kg^−1^.min^−1^ higher in Rural) was greater than the difference between Rural and Urban Europeans (4.0 ml.kg^−1^.min^−1^ higher in Rural). In addition to this interaction effect, VO_2max_ was higher in Rural compared to Urban groups (*p* = 0.0001).

**Table 3:** Fitness, physical activity and dietary intake variables by ethnic group and environment.

	Mapuche		European		*p* value		
	Rural	Urban	Rural	Urban	Ethn	Env	Ethn x Env Interaction
**Physical Activity and fitness variables**							
VO_2max_ (ml.kg.min^−1^)	53.3±12.3	44.7±10.5	45.7±11.8	41.7±9.9	(0.010)	**(0.0001)**	**0.0001**
Sedentary time (min.day^−1^)	499.3±74.8	536.9±98.1	505.9±95.9	546.7±87.2	0.725	**0.0001**	0.929
Light activity (min.day^−1^)	252.4±65.3	236.0±82.3	260.1±95.2	246.8±92.2	0.419	0.149	0.868
Moderate-to-vigorous activity (min.day^−1^)	44.1±30.5	33.5±23.2	41.2±31.4	27.7±16.1	0.508	**0.0001**	0.909
**Dietary intake variables**							
Energy intake (kcal.day^−1^)	3362.1±833	2714.1±710	2392.6±598	2228.3±623	**(0.0001)**	**(0.0001)**	**0.0001**
Carbohydrate intake (g.day^−1^)	461.7±133.4	406.7±119.5	350.1±101.1	318.5±108.6	**0.0001**	**0.0001**	0.099
Fat intake (g.day^−1^)	107.9±42.1	74.4±30.6	61.9±31.8	64.2±27.8	**(0.0001)**	**(0.0001)**	**0.0001**
Protein intake (g.day^−1^)	108.9±50.2	93.4±25.8	98.8±35.6	83.9±32.0	0.034	**0.0001**	0.540
Dietary fibre intake (g.day^−1^)	7.9±4.4	5.38±3.2	4.4±2.63	4.0±1.8	**(0.0001)**	**(0.0001)**	**0.0003**
Alcohol intake (g.day^−1^)	15.3±11.6	6.2±4.8	5.6±7.7	5.6±10.8	**(0.0001)**	**(0.0001)**	**0.0001**

Data are presented as mean ± SD. Statistical analysis was undertaken on age- and sex-adjusted data. *p* values shown are for comparisons of means between Rural Mapuche, Urban Mapuche, Rural European and Urban European groups for main effects of ethnicity (Ethn) and of environment (Env), and the Ethnicity x environment (Ethn x Env) interaction effect: for the main effects, those in parentheses indicate residual main effects in a model with a significant interaction term, while those not in parentheses indicate main effects in a model without the interaction term. Significant *p* values (i.e. *p*<0.004) are shown in bold.

No significant ethnicity x environment interactions were observed for any of the activity variables, but sedentary time was higher (*p* = 0.0001) and moderate-to-vigorous activity time was lower (*p* = 0.0001) in Urban compared to Rural groups.

### Dietary variables for Mapuches and Europeans living in Rural and Urban Environments

Dietary factors differed noticeably between sub-groups (Table 3). Significant ethnicity x environment interactions were observed for energy, fat, dietary fibre and alcohol intakes (all *p*<0.004), indicating that ethnic differences exist for the effects of urbanisation on diet. For all these dietary components the differences between Rural and Urban populations were greater in Mapuches than Europeans. In addition to these interaction effects, Rural participants had higher intakes than Urban participants for all dietary components, and Mapuches had higher intakes than Europeans for all dietary components, except protein (all *p*<0.004).

### Effects of adiposity on HOMA_IR_ in Mapuches and Europeans

**Figure 2:**
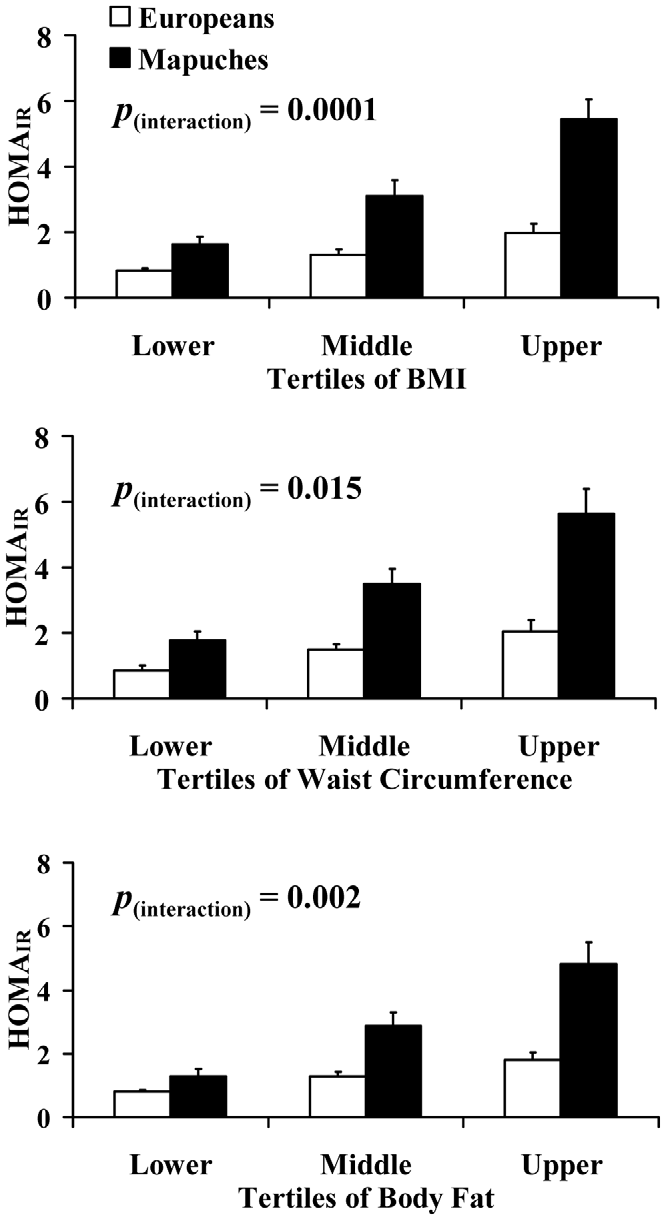
Effects of BMI, waist circumference and body fat on HOMA_IR_ in European and Mapuche participants. Bars show mean ± SEM for all groups. *p*_(interaction)_ describes the ‘tertile x environment’ interaction for HOMA_IR_ after adjustment for potential confounding covariates (tertile cut-points shown in Table S2, see text or Table S3 for list of covariates).

Figure 2 shows the effect of increases in BMI, waist circumference and percentage body fat on HOMA_IR_ in Europeans and Mapuches. There was a significant trend overall for HOMA_IR_ to increase with increasing BMI, waist circumference and percentage body fat (all *p*<0.004, Table S3). In age- and sex-adjusted analyses there were significant ‘ethnicity x BMI tertile’ and ‘ethnicity x body fat tertile’ interactions for HOMA_IR_ (*p*<0.004) with Mapuches experiencing significantly greater increases in HOMA_IR_ with increasing BMI or body fat than Europeans (Figure 2, Table S3). The ‘ethnicity x waist tertile’ interaction followed the same pattern but only achieved borderline statistical significance (*p* = 0.007). Further adjustment for smoking status, environment (rural or urban), socio-economic level and education level did not alter the statistical significance of any of these findings (Table S3).

### Effects of physical activity, sedentary time and fitness on HOMA_IR_ in Mapuches and Europeans

**Figure 3:**
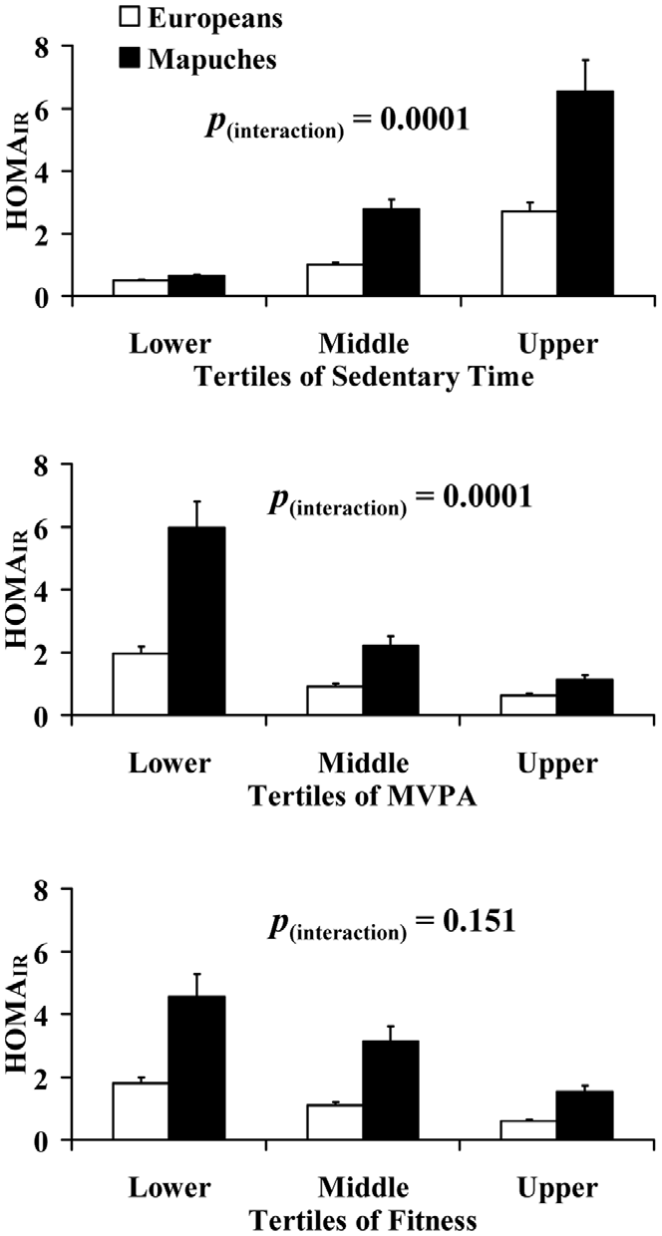
Effects of sedentary time, moderate-to-vigorous physical activity and fitness on HOMA_IR_ in European and Mapuche participants. Bars show mean ± SEM for all groups. *p*_(interaction)_ describes the ‘tertile x environment’ interaction for HOMA_IR_ after adjustment for potential confounding covariates (tertile cut-points shown in Table S2, see text or Table S4 for list of covariates).

Figure 3 shows the effects of increasing amounts of sedentary time, moderate-to-vigorous physical activity (MVPA) and fitness (as measured using VO_2max_) on HOMA_IR_ in Europeans and Mapuches. As for the anthropometric variables described above, there were significant trends for HOMA_IR_ to increase with increasing tertiles of sedentary time, and with decreasing tertiles of MVPA and decreasing tertiles of VO_2max_ (all *p*<0.004, Table S4). Significant ‘ethnicity x sedentary time tertile’ and ‘ethnicity x MVPA tertile’ interactions were also evident, such that Mapuches experienced greater increases in HOMA_IR_ with increasing sedentary time and decreasing MVPA than Europeans in age- and sex-adjusted analyses (all *p*<0.004, Figure 3, Table S4). The ‘ethnicity x sedentary time tertile’ and ‘ethnicity x MVPA tertile’ interactions with HOMA_IR_ were unchanged by further adjustment for: accelerometer wear time, VO_2max_, BMI, waist circumference, percentage body fat, energy intake, smoking status, environment, socio-economic level, education level and MVPA (for the sedentary time model) or sedentary time (for the MVPA model) (both *p*<0.004, Figure 3, Table S4).

### Effects of dietary intake on HOMA_IR_ in Mapuches and Europeans

There was no significant influence of any component of dietary intake on HOMA_IR_.

## Discussion

The main findings of this study were: 1) that Chilean Mapuches are more insulin resistant (as assessed by HOMA_IR_) than Chileans of European descent; 2) urbanisation appears to have a greater effect on insulin resistance in Mapuches than European Chileans – there was a three-fold difference in HOMA_IR_ between Urban and Rural Mapuches, compared to less than a two-fold difference between Urban and Rural European Chileans, with a significant ‘ethnicity x environment’ interaction for this effect; 3) the differences between Urban and Rural populations for physical activity and sedentary time, and percentage body fat were similar between the two ethnic groups, suggesting that the greater difference in HOMA_IR_ between Urban and Rural Mapuches compared to Europeans is not simply the consequence of a larger lifestyle shift between rural and urban environments in the former group; and 4) differences in adiposity, physical activity level and sedentary time had a greater influence on insulin resistance in Mapuches than Europeans.

The findings in relation to insulin resistance are consistent with the pattern observed in Pima Indians. A recent study reported that Pima Indians in the USA with normal glucose tolerance have higher HOMA_IR_ values than Mexican Pimas even after adjustment for differences in BMI (4-fold higher in the unadjusted analysis) or in a BMI-matched sample [27]. In addition, US Pima Indians have been shown to be more insulin resistant than age- and adiposity-matched Americans of European descent [28]. In the present study the greater insulin resistance in the Urban, compared to Rural, Mapuche occurred in the absence of a higher BMI, although percentage body fat was greater in the Urban Mapuche group. However, although percentage body fat was greater in the Urban compared to Rural groups in our sample, the extent of the difference did not differ between the Mapuche and Chileans of European descent, indicating that increasing adiposity, in itself, cannot explain the disproportionately large increase in insulin resistance associated with urbanisation in the Mapuche population.

The present data indicate that increasing adiposity is associated with larger increases in insulin resistance in Mapuches than European Chileans. Moving from the lowest to the highest percentage body fat tertile was associated with an approximate doubling in HOMA_IR_ in European Chileans, but a greater than 3.5-fold increase in HOMA_IR_ in Mapuches. Thus, the data suggest that Mapuche are particularly susceptible to the adverse metabolic effects of obesity. This susceptibility may contribute to the disproportionately large difference in insulin resistance (and reported diabetes prevalence) between Mapuches living in Rural and Urban environments, despite the relatively modest difference in adiposity between these two groups. Similar observations have been made when considering other groups at increased risk of type 2 diabetes. For example, children [29] of South Asian origin exhibit larger increases in insulin resistance with increasing adiposity than White Europeans. Our data do not allow conclusions to be drawn about why adiposity had a more potent effect on insulin resistance in the Mapuche group. Speculatively, this could be due to differences in ectopic and regional fat distribution, in adipocyte size, or in adipose tissue signalling, between the groups, but further study is needed to test this hypothesis.

It is well established that high levels of physical activity [13] and cardiorespiratory fitness [14] are protective against type 2 diabetes and are associated with a favourable metabolic risk profile [30]. Recently, it has also become evident that increasing sedentary time is associated with increased diabetes risk [31], [32] and an adverse metabolic risk profile [33], [34] and that these effects may be independent of time spent in physical activity [32], [34]. Reports have shown that low levels of self-reported physical activity or low cardiorespiratory fitness are associated with insulin resistance in other indigenous groups [35]. The findings here are consistent with these observations, and extend them by using objective measures of physical activity and sedentary time and by revealing that the effects on insulin resistance of a low level of moderate-to-vigorous physical activity, or a high level of sedentary time was greater in the Mapuche than the European Chileans. For example, moving from the lowest to the highest tertile for sedentary time was associated with a 5.5-fold increase in insulin resistance in Chileans of European descent, but a 12.2-fold increase in Mapuches. Thus, the adverse effects of sedentariness and low physical activity appear particularly large in this indigenous group. Again, we cannot draw conclusions about why Mapuches are particularly susceptible to the adverse effects of low physical activity and high sedentary time on insulin resistance. However, it is clear that this effect is not simply mediated by differences in adiposity, fitness, dietary intake, smoking, or socioeconomic level or education between the Mapuche and European groups: statistically adjusting for these potential confounders did not influence the findings. Thus, further studies are needed to address the underlying mechanisms.

There was no significant influence of any dietary component on insulin resistance in either the Mapuche or European populations. This is consistent with other observational [36], [37] and intervention trial [38] data, in populations of European descent, showing no effect of diet on insulin resistance or diabetes risk once the effects of diet on adiposity are accounted for. Thus, despite the difference in a number of dietary variables between rural and urban populations being greater in Mapuches than Europeans, the present data indicate this larger dietary difference does not explain the larger effect of environment on insulin sensitivity in the Mapuches.

Data collection in this study presented a number of logistical challenges. The rural Mapuches generally live in remote areas far from road access or mains electricity. Data collection from some of these villages required transport of all equipment (including generators to provide electricity and centrifuges to spin blood) by horseback from site to site. Given these issues, the study is relatively large, with the final sample including 247 Mapuches and 225 European Chileans. However, we recognise that with a sample of this size our ability to draw strong conclusions about population-level group differences is somewhat limited. A particular strength of the study is the detailed phenotyping of the study populations, particularly with respect to lifestyle measures. Fitness, physical activity and sedentary time were objectively measured, providing greater validity in these measures than would be obtained from self-report questionnaire [39]. Dietary intake was also objectively measured by 7-day weighed food records – the best available method. Although underreporting is a common problem in the measurement of dietary intake [40], [41], this does not appear to have been a major factor here as reported energy intakes (means of ∼2900 kcal per day in men and ∼2500 kcal per day in women) were relatively high. Due to the data collection challenges and sample size, we used HOMA_IR_, rather than a euglycaemic clamp or intra-venous glucose tolerance test, to assess insulin sensitivity. It will be important to seek verification that our conclusions remain valid when insulin sensitivity is assessed using more sophisticated techniques. Because the study is cross-sectional in nature, it is not possible to draw firm conclusions about the causality of associations observed. However, our comprehensive study design, with relatively precise measures of a number of relevant exposure variables, allowed us to control for a number of potential confounding variables in our analyses. Although potential residual confounding effects cannot be excluded, the robustness of our findings to these adjustments supports our conclusions.

While the present findings have been adjusted for a number of potential confounders, it was not possible to collect data on birthweight in our cohort, so we were unable to control for potential effects of early life environment. There is a growing body of evidence that fetal environmental effects – which are reflected in size at birth – can influence adult health, potentially through epigenetic programming mechanisms [42]. These early life effects may interact with adult environment to determine disease risk: for example, it has been reported that low birth size was associated with increased risk of the metabolic syndrome in inactive, but not in active, middle-aged Finnish men [43]. However, a recent study found no differences in birthweights between Chileans of Mapuche and non-Mapuche origin born between 2000 and 2004 [44], although no such data are available for the generations represented in the present cohort. Thus, while it is possible that differences in early life environment could have contributed to the ethnicity x (adult) environment interactions observed in the present study, the data are not available to permit any firm conclusions to be drawn on this issue.

In conclusion, the present study reveals that urbanisation, adiposity, physical activity and sedentary behaviour influence insulin resistance to a greater extent in Chilean Mapuches than in Chileans of European descent. These associations persist after adjustment for a comprehensive range of potential confounding factors. The cross-sectional nature of these data does not allow firm conclusions to be drawn about causality, but the findings highlight the fact that environmental and lifestyle effects on metabolic risk differ between ethnic groups and suggest that further studies into the mechanisms underpinning this effect are needed. This has potential implications both for the design and implementation of lifestyle strategies to reduce metabolic risk in different ethnic groups, and for advancing the understanding of the mechanisms underpinning human insulin resistance.

## Supporting Information

Table S1 Number of participants with valid data by data category.
(DOC)

Table S2 Tertile cut-points for obesity-related phenotypes, physical activity and fitness for men and women.
(DOC)

Table S3 Effects of BMI, waist circumference and body fat on HOMA_IR_ in European and Mapuche men and women in age-sex and fully-adjusted models.
(DOC)

Table S4 Effects of sedentary time, moderate-to-vigorous physical activity and fitness on HOMA_IR_ in European and Mapuche men and women in age-sex and fully-adjusted models.
(DOC)
