# Supplementary material for: Insulin Resistance in Chileans of European and Indigenous Descent: Evidence for an Ethnicity x Environment Interaction
Source: PLoS One. 2011 Sep 8;6(9):e24690. doi: 10.1371/journal.pone.0024690 (PMC3169638; doi:10.1371/journal.pone.0024690)
Supplement: Table S1 — Number of participants with valid data by data category. (DOC) [file pone.0024690.s001.doc]

**Table S1. Number of participants with valid data by data category**

|  | Mapuche | | European | |
| --- | --- | --- | --- | --- |
|  | Rural | Urban | Rural | Urban |
| Age, socioeconomic level, education, smoking, anthropometric data (except body fat) | 123 | 124 | 91 | 134 |
| Percentage body fat and VO_2max_ | 123 | 95 | 91 | 134 |
| Metabolic variables | 85 | 116 | 65 | 96 |
| Dietary intake variables | 99 | 82 | 79 | 115 |
| Physical activity variables | 99 | 73 | 73 | 90 |
