# Supplementary material for: Insulin Resistance in Chileans of European and Indigenous Descent: Evidence for an Ethnicity x Environment Interaction
Source: PLoS One. 2011 Sep 8;6(9):e24690. doi: 10.1371/journal.pone.0024690 (PMC3169638; doi:10.1371/journal.pone.0024690)
Supplement: Table S2 — Tertile cut-points for obesity-related phenotypes, physical activity and fitness for men and women. (DOC) [file pone.0024690.s002.doc]

**Table S2. Tertile cut-points for obesity-related phenotypes, physical activity and fitness for men and women.**

|  | Variables | Tertile | | |
| --- | --- | --- | --- | --- |
| Men |  | Lower | Middle | Upper |
|  | BMI (kg.m^-1^) | < 26.6 | 26.6 - 30.1 | > 30.1 |
|  | Waist Circumference (cm) | < 97.0 | 97.0 - 111.0 | > 111.0 |
|  | Body Fat (%) | < 25.7 | 25.7 - 31.9 | > 31.9 |
|  | Sedentary time (min.day^-1^) | < 466 | 466 - 559 | > 559 |
|  | MVPA (min.day^-1^) | < 21.6 | 21.6 - 51.3 | > 51.3 |
|  | Fitness (VO_2max_, ml.kg.min^-1^) | < 44.6 | 44.6 - 54.9 | > 54.9 |
| Women |  |  |  |  |
|  | BMI (kg.m^-1^) | < 26.8 | 26.8 - 31.1 | > 31.1 |
|  | Waist Circumference (cm) | < 96.0 | 96.0 - 110.5 | > 110.5 |
|  | Body Fat (%) | < 29.2 | 29.2 - 33.9 | > 33.9 |
|  | Sedentary time (min.day^-1^) | < 485 | 485 - 559 | > 559 |
|  | MVPA (min.day^-1^) | < 20.1 | 20.1 - 36.5 | > 36.5 |
|  | Fitness (VO_2max_, ml.kg.min^-1^) | < 36.3 | 36.3 - 47.2 | > 47.2 |
