# Supplementary material for: Insulin Resistance in Chileans of European and Indigenous Descent: Evidence for an Ethnicity x Environment Interaction
Source: PLoS One. 2011 Sep 8;6(9):e24690. doi: 10.1371/journal.pone.0024690 (PMC3169638; doi:10.1371/journal.pone.0024690)
Supplement: Table S3 — Effects of BMI, waist circumference and body fat on HOMAIR in European and Mapuche men and women in age-sex and fully-adjusted models. (DOC) [file pone.0024690.s003.doc]

**Table S3. Effects of BMI, waist circumference and body fat on HOMA_IR_ in European and Mapuche men and women in age-sex and fully-adjusted models.**

| Model Factors | Ethnicity | Tertiles | | | *p* values | | |
| --- | --- | --- | --- | --- | --- | --- | --- |
| Ethnicity  BMI tertile (kg.m^-2^)  Covariates^b^ |  | **Lower** | **Middle** | **Upper** | **Ethn** | **BMI** | **Ethn x BMI Interaction** |
|  | Europeans | 0.83 ± 0.05 | 1.31 ± 0.15 | 1.97 ± 0.26 | **0.0001**^a^ | **0.0001**^a^ | **0.003** ^a^ |
|  | Mapuches | 1.63 ± 0.23 | 3.10 ± 0.48 | 5.44 ± 0.61 | **0.0001**^b^ | **0.0001**^b^ | **0.0001**^b^ |
|  |  |  |  |  |  |  |  |
| Ethnicity  Waist tertile (cm)  Covariates^b^ |  | **Lower** | **Middle** | **Upper** | **Ethn** | **Waist** | **Ethn x Waist Interaction** |
|  | Europeans | 0.85 ± 0.06 | 1.50 ± 0.15 | 2.03 ± 0.36 | **0.0001**^a^ | **0.0001**^a^ | 0.007^a^ |
|  | Mapuches | 1.77 ± 0.27 | 3.49 ± 0.46 | 5.64 ± 0.74 | **0.0001**^b^ | **0.0001**^b^ | 0.015^b^ |
|  |  |  |  |  |  |  |  |
| Ethnicity  Body fat (%)  Covariates^b^ |  | **Lower** | **Middle** | **Upper** | **Ethn** | **Body fat** | **Ethn x Body fat Interaction** |
|  | Europeans | 0.79 ± 0.05 | 1.29 ± 0.12 | 1.80 ± 0.24 | **0.0001**^a^ | **0.0001**^a^ | **0.001**^a^ |
|  | Mapuches | 1.29 ± 0.22 | 2.87 ± 0.41 | 4.82 ± 0.66 | **0.0001**^b^ | **0.0001**^b^ | **0.002** ^b^ |

Data are presented as mean ± SEM for untransformed and age and sex-adjusted data. P values shown are main effect for ethnicity (Ethn), main effect for adiposity factors (BMI, waist, body fat) and ethnicity x adiposity interaction effect in age-adjusted^a^  and fully adjusted models^b^. Significant *p* values (i.e. *p* < 0.004) are shown in bold.

^a^*p* values from models adjusted for age and sex.

^b^*p* values from models adjusted for age, sex, environment (rural or urban), socio-economic level, education level, and smoking status.
