# Supplementary material for: Insulin Resistance in Chileans of European and Indigenous Descent: Evidence for an Ethnicity x Environment Interaction
Source: PLoS One. 2011 Sep 8;6(9):e24690. doi: 10.1371/journal.pone.0024690 (PMC3169638; doi:10.1371/journal.pone.0024690)
Supplement: Table S4 — Effects of sedentary time, moderate-to-vigorous physical activity and fitness on HOMAIR in European and Mapuche men and women in age-sex and fully-adjusted models. (DOC) [file pone.0024690.s004.doc]

**Table S4. Effects of sedentary time, moderate-to-vigorous physical activity and fitness on HOMA_IR_ in European and Mapuche men and women in age-sex and fully-adjusted models.**

| Model Factors | Ethnicity | Tertiles | | | *p* values | | |
| --- | --- | --- | --- | --- | --- | --- | --- |
| Ethnicity  Sedentary tertile (min.day^-1^)  Covariates^b^ |  | **Lower** | **Middle** | **Upper** | **Ethn** | **Sedentary** | **Ethn x Sedentary Interaction** |
|  | Europeans | 0.49 ± 0.03 | 1.01 ± 0.06 | 2.70 ± 0.28 | **0.0001**^a^ | **0.0001**^a^ | **0.0001**^a^ |
|  | Mapuches | 0.65 ± 0.03 | 2.77 ± 0.31 | 7.96 ± 1.13 | **0.0001**^b^ | **0.0001**^b^ | **0.0001**^b^ |
|  |  |  |  |  |  |  |  |
| Ethnicity  MVPA tertile (min.day^-1^)  Covariates^c^ |  | **Lower** | **Middle** | **Upper** | **Ethn** | **MVPA** | **Ethn x MVPA Interaction** |
|  | Europeans | 1.96 ± 0.21 | 0.90 ± 0.09 | 0.63 ± 0.05 | **0.0001**^a^ | **0.0001**^a^ | **0.0001**^a^ |
|  | Mapuches | 5.95 ± 0.85 | 2.20 ± 0.32 | 1.12 ± 0.14 | **0.0001**^c^ | **0.0001**^c^ | **0.0001**^c^ |
|  |  |  |  |  |  |  |  |
| Ethnicity  Fitness tertile (VO_2max_, ml.kg.min^-1^)  Covariates^d^ |  | **Lower** | **Middle** | **Upper** | **Ethn** | **Fitness** | **Ethn x Fitness Interaction** |
|  | Europeans | 1.80 ± 0.18 | 1.10 ± 0.08 | 0.59 ± 0.05 | **0.0001**^a^ | **0.0001**^a^ | 0.050^a^ |
|  | Mapuches | 4.55 ± 0.70 | 3.12 ± 0.49 | 1.53 ± 0.18 | **0.0001**^d^ | **0.0001**^d^ | 0.151^d^ |

Data are presented as mean ± SEM for untransformed and age and sex-adjusted data. P values shown are main effect for ethnicity (Ethn), main effect for fitness/activity factors (sedentary time, MVPA, fitness) and ethnicity x fitness/activity interaction effect in age-adjusted**^a^** and fully adjusted models^b,c,d^. Significant *p* values (i.e. *p* < 0.004) are shown in bold.

^a^*p* values from models adjusted for age and sex.

^b^*p* values from models adjusted for age, sex, environment (rural or urban), socio-economic level, education level, smoking status, BMI, waist, body fat, accelerometer wear time, MVPA, fitness and energy intake.

^c^*p* values from models adjusted for age, sex, environment, socio-economic level, education level, smoking status, BMI, waist, body fat, accelerometer wear time, sedentary time, fitness and energy intake.

^d^*p* values from models adjusted for age, sex, environment, socio-economic level, education level, smoking status, BMI, waist, body fat, accelerometer wear time, sedentary time, MVPA and energy intake.
